# *N*-(4-Amino­phen­yl)-4-methylbenzene­sulfonamide

**DOI:** 10.1107/S1600536811036191

**Published:** 2011-09-30

**Authors:** Jeveria Rehman, Ejaz, Islam Ullah Khan, William T. A. Harrison

**Affiliations:** a Materials Chemistry Laboratry, Department of Chemistry, GC University, Lahore 54000, Pakistan; b Department of Chemistry, University of Aberdeen, Meston Walk, Aberdeen AB24 3UE, Scotland

## Abstract

The title compound, C_13_H_14_N_2_O_2_S, crystallized with two independent mol­ecules in the asymmetric unit. They both have V-shaped conformations: the dihedral angles between their benzene rings are identical [45.86 (13)°] and their C—S—N—C torsion angles are similar [67.9 (3) and 70.2 (3)°]. In the crystal, the mol­ecules are linked by N—H⋯O and N—H⋯N hydrogen bonds, generating a three-dimensional network.

## Related literature

For related structures and background to sulfonamides, see: Xing & Zeng (2005 ▶); Gelbrich *et al.* (2007 ▶); Khan *et al.* (2010 ▶, 2011 ▶).
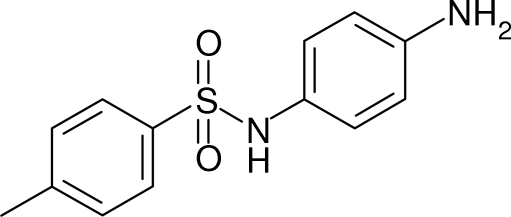


## Experimental

### 

#### Crystal data

C_13_H_14_N_2_O_2_S
*M* *_r_* = 262.32
Orthorhombic, 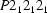

*a* = 5.0598 (3) Å
*b* = 14.7702 (11) Å
*c* = 35.026 (2) Å
*V* = 2617.7 (3) Å^3^
*Z* = 8
Mo *K*α radiation
μ = 0.24 mm^−1^
*T* = 296 K
0.41 × 0.35 × 0.20 mm

#### Data collection

Bruker APEXII CCD diffractometer
Absorption correction: multi-scan (*SADABS*; Bruker, 2001 ▶) *T*_min_ = 0.907, *T*_max_ = 0.953
14160 measured reflections
6049 independent reflections
3138 reflections with *I* > 2σ(*I*)
*R*_int_ = 0.048

#### Refinement

*R*[*F* ^2^ > 2σ(*F* ^2^)] = 0.058
*wR*(*F* ^2^) = 0.122
*S* = 0.98
6049 reflections
345 parameters
H atoms treated by a mixture of independent and constrained refinement
Δρ_max_ = 0.19 e Å^−3^
Δρ_min_ = −0.23 e Å^−3^
Absolute structure: Flack (1983 ▶), 2266 Friedel pairs
Flack parameter: −0.03 (8)

### 

Data collection: *APEX2* (Bruker, 2007 ▶); cell refinement: *SAINT* (Bruker, 2007 ▶); data reduction: *SAINT*; program(s) used to solve structure: *SHELXS97* (Sheldrick, 2008 ▶); program(s) used to refine structure: *SHELXL97* (Sheldrick, 2008 ▶); molecular graphics: *ORTEP-3* (Farrugia, 1997 ▶); software used to prepare material for publication: *SHELXL97*.

## Supplementary Material

Crystal structure: contains datablock(s) I, global. DOI: 10.1107/S1600536811036191/su2300sup1.cif

Structure factors: contains datablock(s) I. DOI: 10.1107/S1600536811036191/su2300Isup2.hkl

Supplementary material file. DOI: 10.1107/S1600536811036191/su2300Isup3.cml

Additional supplementary materials:  crystallographic information; 3D view; checkCIF report

## Figures and Tables

**Table 1:** Hydrogen-bond geometry (Å, °)

*D*—H⋯*A*	*D*—H	H⋯*A*	*D*⋯*A*	*D*—H⋯*A*
N1—H1*N*⋯O2^i^	0.70 (3)	2.33 (4)	2.996 (4)	161 (5)
N2—H2*N*⋯O4^ii^	0.88 (4)	2.18 (4)	3.052 (4)	172 (4)
N2—H3*N*⋯N4^iii^	0.97 (4)	2.28 (4)	3.191 (5)	155 (4)
N3—H4*N*⋯O3^i^	0.79 (3)	2.17 (3)	2.957 (3)	170 (3)
N4—H5*N*⋯N2^iv^	0.85 (4)	2.45 (4)	3.241 (5)	155 (4)
N4—H6*N*⋯O1^v^	0.86 (4)	2.47 (4)	3.323 (5)	171 (4)

Symmetry codes: (i) 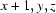; (ii) 
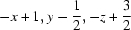
; (iii) 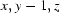; (iv) 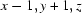; (v) 
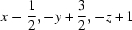
.

**Fig. 1.**
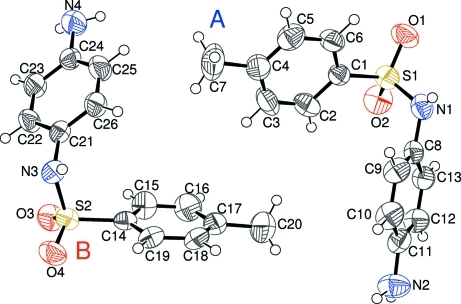
The molecular structure of the two independent molecules (A and B) of compound (I), showing the numbering scheme and displacement ellipsoids drawn at the 50% probability level.

**Fig. 2.**
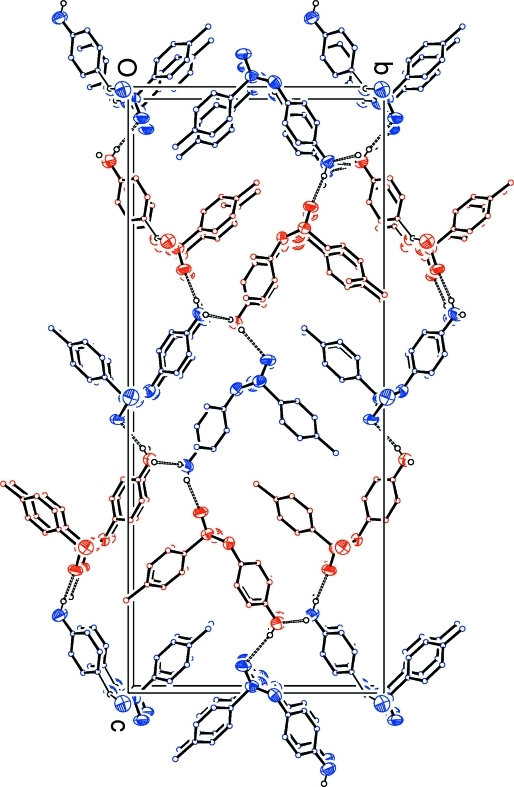
The crystal packing of compound (I), viewed along the a-axis, with the N-H···O and N-H···N hydrogen bonds (dashed lines; see Table 1 for details). Molecule A is shown in blue and molecule A in red. All C-bound H atoms have been omitted for clarity.

## supplementary crystallographic information

### Comment

As part of our ongoing structural studies of sulfonamides (Khan *et al.*, 2011), the synthesis and structure of the title compound, (I) (Fig. 1), are now described. Related stuctures with different substituents replacing the *para*-amino group in (I) include 4-methyl-*N*-(4-nitrophenyl)benzenesulfonamide, (II), (Xing and Zeng, 2005) and 4-methyl-*N*-(4-methylphenyl)benzenesulfonamide, (III), (Khan *et al.*, 2010). Also worthy of mention is the remarkable sudy of Gelbrich *et al.* (2007), who made a systematic structural comparison of no fewer than 133 crystal structures of 4,4'-disubstituted benzenesulfonamidobenzenes. However, no compounds with an amino substituent were incorporated into their survey.

There are two independent molecules (A containing S1 and B containing S2) in the asymmetric unit of (I), as shown in Fig. 1. They have similar V-shaped conformations and indeed the dihedral angles between their benzene rings are identical [45.86 (13)°]. Their C—S—N—C torsion angles [67.9 (3)° for A and 70.2 (3)° for B] are also similar. The bond angle sums for N1 and N3 [349.4 and 344.1°, respectively] indicate a clear tendancy towards pyramidal geometry for the nitrogen atoms. Otherwise, their bond lengths and angles are similar to those seen in (II) and (III). Values for the inter-ring dihedral angle and the C—S—N—C torsion angle are 86.1 (1) and 65.85 (13)°, respectively, in (II), and 70.53 (10) and -60.71 (18)°, respectively, in (III), indicating that (I), (II) and (III) have significantly different conformations.

In the crystal of (I), the molecules are linked by N—H···O and N—H···N hydrogen bonds (Table 1). Both the sulfonamide NH groups make intermolecular hydrogen bonds to the sulfonamide O-atom acceptors such that separate [100] C(4) chains of A and B are generated, with adjacent molecules related only by a unit-cell translation. The amine groups each form an N—H···N and an N—H···O link. The former bonds lead to distinctive [100] —N—H···N—H···N— C(2) chains of alternating A and B molecules, and serve to link the C(4) chains into a sheet. The latter bonds generate [001] chains of alternating A and B molecules and overall a three-dimensional array is generated (Fig. 2).

Because of the presence of the —NH_2_ group, the hydrogen bonding patterns in (I) are not expected to show close similarities to those of the Gelbrich *et al.* (2007) study. There, the sulfonamide —NH— group was the only possible donor and the structure of (I) does not appear to match any of the groups of structures described by these workers.

### Experimental

*p*-Toluene sulfonyl chloride (2 mmol, 0.3813 g) was added to *p*-phenylenediamine (1 mmol, 0.108 g) in distilled water (20 ml) in a round-bottom flask (100 ml). The suspension was stirred for 10 h at room temperature while keeping the pH between 8–9 with sodium carbonate solution (3%). The light brown precipitate formed was filtered, washed with distilled water and dried. Light brown needles of (I) were grown from methanol.

### Refinement

The N-bound H-atoms were located in difference Fourier maps and their positions were freely refined with the constraint *U*_iso_(H) = 1.2*U*_eq_(N). The C-bound H-atoms were placed in calculated positions treated as riding atoms: C-H = 0.93 and 0.96 Å for CH and CH_3_ H-atoms, respectively, with *U*_iso_(H) = k × *U*_eq_(C), where k = 1.5 for CH_3_ H-atoms and k = 1.2 for all other H-atoms.

### Crystal data

**Table:**

C_13_H_14_N_2_O_2_S	*F*(000) = 1104
*M**_r_* = 262.32	*D*_x_ = 1.331 Mg m^−^^3^
Orthorhombic, *P*2_1_2_1_2_1_	Mo *K*α radiation, λ = 0.71073 Å
Hall symbol: P 2ac 2ab	Cell parameters from 2101 reflections
*a* = 5.0598 (3) Å	θ = 2.3–21.0°
*b* = 14.7702 (11) Å	µ = 0.24 mm^−^^1^
*c* = 35.026 (2) Å	*T* = 296 K
*V* = 2617.7 (3) Å^3^	Cut needle, brown
*Z* = 8	0.41 × 0.35 × 0.20 mm

### Data collection

**Table:**

Bruker APEXII CCD diffractometer	6049 independent reflections
Radiation source: fine-focus sealed tube	3138 reflections with *I* > 2σ(*I*)
graphite	*R*_int_ = 0.048
ω scans	θ_max_ = 28.3°, θ_min_ = 1.2°
Absorption correction: multi-scan (*SADABS*; Bruker, 2001)	*h* = −6→6
*T*_min_ = 0.907, *T*_max_ = 0.953	*k* = −18→19
14160 measured reflections	*l* = −42→44

### Refinement

**Table:**

Refinement on *F*^2^	Secondary atom site location: difference Fourier map
Least-squares matrix: full	Hydrogen site location: inferred from neighbouring sites
*R*[*F*^2^ > 2σ(*F*^2^)] = 0.058	H atoms treated by a mixture of independent and constrained refinement
*wR*(*F*^2^) = 0.122	*w* = 1/[σ^2^(*F*_o_^2^) + (0.0447*P*)^2^] where *P* = (*F*_o_^2^ + 2*F*_c_^2^)/3
*S* = 0.98	(Δ/σ)_max_ = 0.003
6049 reflections	Δρ_max_ = 0.19 e Å^−^^3^
345 parameters	Δρ_min_ = −0.23 e Å^−^^3^
0 restraints	Absolute structure: Flack (1983), 2266 Friedel pairs
Primary atom site location: structure-invariant direct methods	Flack parameter: −0.03 (8)

### Special details

**Table:**

Geometry. All e.s.d.'s (except the e.s.d. in the dihedral angle between two l.s. planes) are estimated using the full covariance matrix. The cell e.s.d.'s are taken into account individually in the estimation of e.s.d.'s in distances, angles and torsion angles; correlations between e.s.d.'s in cell parameters are only used when they are defined by crystal symmetry. An approximate (isotropic) treatment of cell e.s.d.'s is used for estimating e.s.d.'s involving l.s. planes.
Refinement. Refinement of *F*^2^ against ALL reflections. The weighted *R*-factor *wR* and goodness of fit *S* are based on *F*^2^, conventional *R*-factors *R* are based on *F*, with *F* set to zero for negative *F*^2^. The threshold expression of *F*^2^ > σ(*F*^2^) is used only for calculating *R*-factors(gt) *etc*. and is not relevant to the choice of reflections for refinement. *R*-factors based on *F*^2^ are statistically about twice as large as those based on *F*, and *R*- factors based on ALL data will be even larger.

### Fractional atomic coordinates and isotropic or equivalent isotropic displacement parameters (Å^2^)

**Table:**

	*x*	*y*	*z*	*U*_iso_*/*U*_eq_	
C1	0.7219 (6)	0.5980 (2)	0.52219 (9)	0.0461 (9)	
C2	0.5551 (7)	0.6031 (3)	0.55343 (11)	0.0676 (11)	
H2	0.4207	0.5608	0.5565	0.081*	
C3	0.5892 (9)	0.6712 (3)	0.57994 (11)	0.0745 (13)	
H3	0.4774	0.6735	0.6010	0.089*	
C4	0.7816 (9)	0.7354 (3)	0.57630 (11)	0.0650 (11)	
C5	0.9476 (8)	0.7288 (3)	0.54593 (11)	0.0672 (11)	
H5	1.0827	0.7710	0.5433	0.081*	
C6	0.9217 (7)	0.6609 (3)	0.51874 (10)	0.0577 (10)	
H6	1.0386	0.6579	0.4983	0.069*	
C7	0.8067 (12)	0.8109 (3)	0.60412 (11)	0.1064 (17)	
H7A	0.7568	0.8667	0.5921	0.160*	
H7B	0.9864	0.8150	0.6127	0.160*	
H7C	0.6929	0.7998	0.6256	0.160*	
C8	0.7725 (7)	0.3749 (2)	0.53228 (10)	0.0470 (9)	
C9	0.8953 (7)	0.3861 (3)	0.56689 (11)	0.0588 (10)	
H9	1.0308	0.4282	0.5695	0.071*	
C10	0.8171 (9)	0.3350 (3)	0.59756 (11)	0.0667 (11)	
H10	0.9024	0.3423	0.6209	0.080*	
C11	0.6143 (7)	0.2728 (3)	0.59452 (11)	0.0573 (10)	
C12	0.4958 (7)	0.2624 (3)	0.55924 (10)	0.0581 (10)	
H12	0.3598	0.2206	0.5564	0.070*	
C13	0.5747 (7)	0.3124 (3)	0.52862 (10)	0.0521 (9)	
H13	0.4935	0.3040	0.5051	0.062*	
S1	0.67974 (18)	0.51473 (7)	0.48702 (3)	0.0565 (3)	
N1	0.8513 (6)	0.4261 (2)	0.49950 (10)	0.0574 (9)	
H1N	0.988 (7)	0.429 (3)	0.4975 (11)	0.069*	
N2	0.5408 (8)	0.2184 (3)	0.62512 (10)	0.0775 (11)	
H2N	0.589 (8)	0.235 (3)	0.6483 (10)	0.093*	
H3N	0.362 (8)	0.195 (3)	0.6230 (10)	0.093*	
O1	0.7922 (5)	0.54737 (18)	0.45226 (7)	0.0748 (8)	
O2	0.4094 (4)	0.48778 (19)	0.48757 (8)	0.0759 (8)	
C14	0.1982 (6)	0.7212 (2)	0.73078 (9)	0.0433 (8)	
C15	0.0384 (8)	0.7105 (3)	0.69949 (11)	0.0661 (11)	
H15	−0.0987	0.7509	0.6947	0.079*	
C16	0.0848 (9)	0.6391 (3)	0.67542 (12)	0.0760 (13)	
H16	−0.0247	0.6315	0.6543	0.091*	
C17	0.2854 (9)	0.5789 (3)	0.68115 (11)	0.0652 (11)	
C18	0.4447 (8)	0.5923 (3)	0.71206 (11)	0.0625 (11)	
H18	0.5846	0.5527	0.7163	0.075*	
C19	0.4044 (6)	0.6623 (2)	0.73690 (11)	0.0518 (9)	
H19	0.5157	0.6701	0.7578	0.062*	
C20	0.3247 (11)	0.4991 (3)	0.65535 (13)	0.1056 (17)	
H20A	0.4187	0.4525	0.6688	0.158*	
H20B	0.4248	0.5173	0.6334	0.158*	
H20C	0.1559	0.4763	0.6473	0.158*	
C21	0.2515 (6)	0.9433 (2)	0.71400 (10)	0.0434 (9)	
C22	0.0559 (7)	1.0077 (3)	0.71480 (10)	0.0551 (10)	
H22	−0.0298	1.0205	0.7377	0.066*	
C23	−0.0137 (7)	1.0527 (3)	0.68273 (10)	0.0581 (10)	
H23	−0.1487	1.0954	0.6838	0.070*	
C24	0.1128 (7)	1.0364 (2)	0.64826 (10)	0.0517 (9)	
C25	0.3122 (8)	0.9737 (3)	0.64794 (11)	0.0659 (11)	
H25	0.4034	0.9627	0.6254	0.079*	
C26	0.3792 (7)	0.9273 (2)	0.68016 (10)	0.0563 (10)	
H26	0.5131	0.8842	0.6792	0.068*	
S2	0.14355 (16)	0.81143 (6)	0.76258 (3)	0.0478 (3)	
N3	0.3200 (5)	0.89631 (19)	0.74834 (8)	0.0448 (7)	
H4N	0.474 (6)	0.886 (2)	0.7494 (9)	0.054*	
N4	0.0471 (8)	1.0859 (3)	0.61552 (10)	0.0748 (11)	
H5N	−0.110 (8)	1.104 (3)	0.6191 (12)	0.090*	
H6N	0.099 (8)	1.055 (3)	0.5963 (11)	0.090*	
O3	−0.1258 (4)	0.83822 (17)	0.75914 (7)	0.0647 (7)	
O4	0.2457 (5)	0.78643 (19)	0.79860 (6)	0.0668 (8)	

### Atomic displacement parameters (Å^2^)

**Table:**

	*U*^11^	*U*^22^	*U*^33^	*U*^12^	*U*^13^	*U*^23^
C1	0.0446 (19)	0.049 (2)	0.045 (2)	0.0025 (18)	0.0005 (17)	0.0031 (17)
C2	0.057 (2)	0.082 (3)	0.064 (3)	−0.005 (2)	0.016 (2)	0.006 (3)
C3	0.081 (3)	0.091 (4)	0.052 (3)	0.012 (3)	0.021 (2)	−0.010 (3)
C4	0.081 (3)	0.062 (3)	0.053 (3)	0.016 (3)	−0.001 (2)	0.004 (2)
C5	0.074 (3)	0.057 (3)	0.070 (3)	−0.009 (2)	−0.012 (2)	−0.002 (2)
C6	0.056 (2)	0.065 (3)	0.052 (2)	−0.007 (2)	0.0099 (19)	0.003 (2)
C7	0.164 (5)	0.076 (3)	0.079 (3)	0.030 (4)	−0.005 (3)	−0.028 (3)
C8	0.042 (2)	0.048 (2)	0.051 (2)	0.0038 (18)	0.0044 (18)	−0.0066 (19)
C9	0.057 (2)	0.053 (3)	0.066 (3)	−0.010 (2)	−0.013 (2)	−0.002 (2)
C10	0.080 (3)	0.060 (3)	0.061 (3)	−0.010 (2)	−0.026 (2)	−0.008 (2)
C11	0.065 (3)	0.046 (2)	0.061 (3)	0.002 (2)	−0.010 (2)	−0.003 (2)
C12	0.062 (2)	0.056 (3)	0.057 (2)	−0.014 (2)	−0.010 (2)	−0.003 (2)
C13	0.055 (2)	0.052 (2)	0.049 (2)	−0.007 (2)	−0.0062 (17)	−0.007 (2)
S1	0.0551 (6)	0.0642 (7)	0.0502 (6)	−0.0055 (5)	−0.0044 (5)	−0.0030 (5)
N1	0.0490 (18)	0.062 (2)	0.061 (2)	−0.001 (2)	0.0063 (19)	−0.0103 (17)
N2	0.092 (3)	0.080 (3)	0.061 (2)	−0.018 (2)	−0.016 (2)	0.015 (2)
O1	0.098 (2)	0.087 (2)	0.0397 (15)	−0.0100 (18)	0.0080 (15)	0.0010 (14)
O2	0.0483 (14)	0.088 (2)	0.092 (2)	−0.0104 (15)	−0.0146 (14)	−0.0059 (18)
C14	0.0405 (18)	0.044 (2)	0.046 (2)	−0.0038 (17)	0.0029 (17)	0.0089 (17)
C15	0.059 (2)	0.071 (3)	0.068 (3)	0.007 (2)	−0.010 (2)	−0.006 (2)
C16	0.084 (3)	0.079 (3)	0.065 (3)	−0.008 (3)	−0.017 (2)	−0.006 (3)
C17	0.089 (3)	0.050 (3)	0.057 (3)	−0.014 (3)	0.016 (3)	−0.003 (2)
C18	0.078 (3)	0.040 (2)	0.070 (3)	0.005 (2)	0.008 (2)	0.011 (2)
C19	0.052 (2)	0.044 (2)	0.059 (2)	−0.0008 (19)	−0.0048 (19)	0.010 (2)
C20	0.163 (5)	0.071 (3)	0.083 (3)	−0.004 (4)	0.010 (3)	−0.021 (3)
C21	0.0367 (19)	0.039 (2)	0.054 (2)	0.0010 (17)	0.0005 (17)	−0.0027 (18)
C22	0.062 (2)	0.058 (3)	0.046 (2)	0.021 (2)	0.0102 (18)	0.003 (2)
C23	0.060 (2)	0.055 (3)	0.059 (3)	0.021 (2)	0.003 (2)	0.002 (2)
C24	0.065 (2)	0.044 (2)	0.046 (2)	0.002 (2)	−0.004 (2)	0.0006 (19)
C25	0.072 (3)	0.067 (3)	0.059 (3)	0.017 (3)	0.017 (2)	0.000 (2)
C26	0.055 (2)	0.053 (2)	0.061 (3)	0.018 (2)	0.007 (2)	0.000 (2)
S2	0.0404 (5)	0.0560 (6)	0.0469 (6)	0.0052 (5)	0.0017 (4)	0.0051 (5)
N3	0.0351 (14)	0.0449 (17)	0.0546 (18)	0.0059 (15)	−0.0076 (14)	0.0033 (15)
N4	0.092 (3)	0.071 (3)	0.061 (2)	0.009 (2)	0.004 (2)	0.004 (2)
O3	0.0423 (14)	0.0779 (19)	0.0739 (17)	0.0104 (13)	0.0112 (13)	0.0022 (15)
O4	0.0828 (19)	0.077 (2)	0.0406 (14)	0.0086 (15)	−0.0042 (13)	0.0097 (13)

### Geometric parameters (Å, °)

**Table:**

C1—C6	1.378 (4)	C14—C15	1.371 (4)
C1—C2	1.384 (4)	C14—C19	1.375 (4)
C1—S1	1.754 (4)	C14—S2	1.759 (3)
C2—C3	1.380 (5)	C15—C16	1.370 (5)
C2—H2	0.9300	C15—H15	0.9300
C3—C4	1.364 (5)	C16—C17	1.365 (6)
C3—H3	0.9300	C16—H16	0.9300
C4—C5	1.359 (5)	C17—C18	1.364 (5)
C4—C7	1.487 (5)	C17—C20	1.498 (6)
C5—C6	1.389 (5)	C18—C19	1.367 (5)
C5—H5	0.9300	C18—H18	0.9300
C6—H6	0.9300	C19—H19	0.9300
C7—H7A	0.9600	C20—H20A	0.9600
C7—H7B	0.9600	C20—H20B	0.9600
C7—H7C	0.9600	C20—H20C	0.9600
C8—C13	1.368 (4)	C21—C26	1.371 (4)
C8—C9	1.372 (4)	C21—C22	1.373 (4)
C8—N1	1.431 (5)	C21—N3	1.431 (4)
C9—C10	1.372 (5)	C22—C23	1.352 (4)
C9—H9	0.9300	C22—H22	0.9300
C10—C11	1.381 (5)	C23—C24	1.388 (4)
C10—H10	0.9300	C23—H23	0.9300
C11—C12	1.382 (4)	C24—C25	1.369 (5)
C11—N2	1.391 (5)	C24—N4	1.400 (5)
C12—C13	1.362 (4)	C25—C26	1.363 (5)
C12—H12	0.9300	C25—H25	0.9300
C13—H13	0.9300	C26—H26	0.9300
S1—O2	1.425 (2)	S2—O4	1.413 (2)
S1—O1	1.428 (3)	S2—O3	1.424 (2)
S1—N1	1.630 (4)	S2—N3	1.618 (3)
N1—H1N	0.70 (3)	N3—H4N	0.79 (3)
N2—H2N	0.88 (4)	N4—H5N	0.85 (4)
N2—H3N	0.97 (4)	N4—H6N	0.86 (4)
			
C6—C1—C2	118.7 (3)	C15—C14—C19	120.0 (3)
C6—C1—S1	120.0 (3)	C15—C14—S2	120.0 (3)
C2—C1—S1	121.3 (3)	C19—C14—S2	120.0 (3)
C3—C2—C1	119.7 (4)	C16—C15—C14	118.6 (4)
C3—C2—H2	120.2	C16—C15—H15	120.7
C1—C2—H2	120.2	C14—C15—H15	120.7
C4—C3—C2	122.2 (4)	C17—C16—C15	122.6 (4)
C4—C3—H3	118.9	C17—C16—H16	118.7
C2—C3—H3	118.9	C15—C16—H16	118.7
C5—C4—C3	117.7 (4)	C18—C17—C16	117.5 (4)
C5—C4—C7	120.9 (4)	C18—C17—C20	121.0 (5)
C3—C4—C7	121.4 (4)	C16—C17—C20	121.5 (4)
C4—C5—C6	122.0 (4)	C17—C18—C19	121.8 (4)
C4—C5—H5	119.0	C17—C18—H18	119.1
C6—C5—H5	119.0	C19—C18—H18	119.1
C1—C6—C5	119.7 (3)	C18—C19—C14	119.5 (4)
C1—C6—H6	120.1	C18—C19—H19	120.2
C5—C6—H6	120.1	C14—C19—H19	120.2
C4—C7—H7A	109.5	C17—C20—H20A	109.5
C4—C7—H7B	109.5	C17—C20—H20B	109.5
H7A—C7—H7B	109.5	H20A—C20—H20B	109.5
C4—C7—H7C	109.5	C17—C20—H20C	109.5
H7A—C7—H7C	109.5	H20A—C20—H20C	109.5
H7B—C7—H7C	109.5	H20B—C20—H20C	109.5
C13—C8—C9	119.7 (3)	C26—C21—C22	118.5 (3)
C13—C8—N1	119.0 (3)	C26—C21—N3	121.9 (3)
C9—C8—N1	121.2 (3)	C22—C21—N3	119.6 (3)
C10—C9—C8	119.6 (4)	C23—C22—C21	120.8 (3)
C10—C9—H9	120.2	C23—C22—H22	119.6
C8—C9—H9	120.2	C21—C22—H22	119.6
C9—C10—C11	121.3 (3)	C22—C23—C24	121.1 (3)
C9—C10—H10	119.3	C22—C23—H23	119.4
C11—C10—H10	119.3	C24—C23—H23	119.4
C10—C11—C12	117.7 (4)	C25—C24—C23	117.7 (3)
C10—C11—N2	121.6 (4)	C25—C24—N4	121.4 (4)
C12—C11—N2	120.6 (4)	C23—C24—N4	120.8 (4)
C13—C12—C11	121.1 (4)	C26—C25—C24	121.1 (3)
C13—C12—H12	119.4	C26—C25—H25	119.4
C11—C12—H12	119.4	C24—C25—H25	119.4
C12—C13—C8	120.4 (3)	C25—C26—C21	120.8 (3)
C12—C13—H13	119.8	C25—C26—H26	119.6
C8—C13—H13	119.8	C21—C26—H26	119.6
O2—S1—O1	119.24 (18)	O4—S2—O3	119.89 (16)
O2—S1—N1	106.46 (18)	O4—S2—N3	106.01 (16)
O1—S1—N1	106.72 (17)	O3—S2—N3	106.66 (16)
O2—S1—C1	107.65 (17)	O4—S2—C14	108.04 (16)
O1—S1—C1	108.28 (17)	O3—S2—C14	107.91 (17)
N1—S1—C1	108.05 (16)	N3—S2—C14	107.78 (15)
C8—N1—S1	119.4 (2)	C21—N3—S2	120.1 (2)
C8—N1—H1N	113 (4)	C21—N3—H4N	112 (2)
S1—N1—H1N	117 (4)	S2—N3—H4N	112 (3)
C11—N2—H2N	119 (3)	C24—N4—H5N	106 (3)
C11—N2—H3N	113 (2)	C24—N4—H6N	107 (3)
H2N—N2—H3N	115 (4)	H5N—N4—H6N	125 (4)
			
C6—C1—C2—C3	1.0 (5)	C19—C14—C15—C16	1.7 (5)
S1—C1—C2—C3	−178.0 (3)	S2—C14—C15—C16	−179.5 (3)
C1—C2—C3—C4	0.8 (6)	C14—C15—C16—C17	−0.7 (6)
C2—C3—C4—C5	−2.1 (6)	C15—C16—C17—C18	−0.7 (6)
C2—C3—C4—C7	176.9 (4)	C15—C16—C17—C20	177.3 (4)
C3—C4—C5—C6	1.5 (6)	C16—C17—C18—C19	1.0 (6)
C7—C4—C5—C6	−177.4 (4)	C20—C17—C18—C19	−177.0 (4)
C2—C1—C6—C5	−1.5 (5)	C17—C18—C19—C14	0.0 (5)
S1—C1—C6—C5	177.5 (3)	C15—C14—C19—C18	−1.4 (5)
C4—C5—C6—C1	0.2 (6)	S2—C14—C19—C18	179.8 (3)
C13—C8—C9—C10	−0.5 (5)	C26—C21—C22—C23	1.6 (5)
N1—C8—C9—C10	−179.1 (3)	N3—C21—C22—C23	−179.6 (3)
C8—C9—C10—C11	−0.8 (6)	C21—C22—C23—C24	−1.0 (6)
C9—C10—C11—C12	1.3 (6)	C22—C23—C24—C25	−0.6 (6)
C9—C10—C11—N2	177.6 (4)	C22—C23—C24—N4	−177.4 (4)
C10—C11—C12—C13	−0.6 (5)	C23—C24—C25—C26	1.7 (6)
N2—C11—C12—C13	−176.9 (4)	N4—C24—C25—C26	178.5 (4)
C11—C12—C13—C8	−0.6 (5)	C24—C25—C26—C21	−1.2 (6)
C9—C8—C13—C12	1.2 (5)	C22—C21—C26—C25	−0.5 (5)
N1—C8—C13—C12	179.8 (3)	N3—C21—C26—C25	−179.2 (3)
C6—C1—S1—O2	−154.1 (3)	C15—C14—S2—O4	154.8 (3)
C2—C1—S1—O2	24.9 (3)	C19—C14—S2—O4	−26.5 (3)
C6—C1—S1—O1	−23.9 (3)	C15—C14—S2—O3	23.8 (3)
C2—C1—S1—O1	155.1 (3)	C19—C14—S2—O3	−157.5 (3)
C6—C1—S1—N1	91.3 (3)	C15—C14—S2—N3	−91.1 (3)
C2—C1—S1—N1	−89.7 (3)	C19—C14—S2—N3	87.7 (3)
C13—C8—N1—S1	81.6 (4)	C26—C21—N3—S2	−100.0 (4)
C9—C8—N1—S1	−99.8 (4)	C22—C21—N3—S2	81.2 (4)
O2—S1—N1—C8	−47.4 (3)	O4—S2—N3—C21	−174.3 (2)
O1—S1—N1—C8	−175.8 (3)	O3—S2—N3—C21	−45.5 (3)
C1—S1—N1—C8	67.9 (3)	C14—S2—N3—C21	70.2 (3)

### Hydrogen-bond geometry (Å, °)

**Table:**

*D*—H···*A*	*D*—H	H···*A*	*D*···*A*	*D*—H···*A*
N1—H1N···O2^i^	0.70 (3)	2.33 (4)	2.996 (4)	161 (5)
N2—H2N···O4^ii^	0.88 (4)	2.18 (4)	3.052 (4)	172 (4)
N2—H3N···N4^iii^	0.97 (4)	2.28 (4)	3.191 (5)	155 (4)
N3—H4N···O3^i^	0.79 (3)	2.17 (3)	2.957 (3)	170 (3)
N4—H5N···N2^iv^	0.85 (4)	2.45 (4)	3.241 (5)	155 (4)
N4—H6N···O1^v^	0.86 (4)	2.47 (4)	3.323 (5)	171 (4)

Symmetry codes: (i) *x*+1, *y*, *z*; (ii) −*x*+1, *y*−1/2, −*z*+3/2; (iii) *x*, *y*−1, *z*; (iv) *x*−1, *y*+1, *z*; (v) *x*−1/2, −*y*+3/2, −*z*+1.
